# Epidemic strain YC2014 of porcine epidemic diarrhea virus could provide piglets against homologous challenge

**DOI:** 10.1186/s12985-016-0529-z

**Published:** 2016-04-22

**Authors:** Huixing Lin, Lei Chen, Lu Gao, Xiaomin Yuan, Zhe Ma, Hongjie Fan

**Affiliations:** College of Veterinary Medicine, Nanjing Agricultural University, Nanjing, China; Jiangsu Co-innovation Center for Prevention and Control of Important Animal Infectious Diseases and Zoonoses, Yangzhou, China

**Keywords:** Porcine epidemic diarrhea virus, Isolation, Immune protective efficiency

## Abstract

**Background:**

Porcine epidemic diarrhea virus (PEDV) is the main causative agent of porcine epidemic diarrhea (PED). Since December 2010, a large-scale outbreak of diarrhea has been observed in swine farms in China. Accumulated evidence indicates that this large-scale outbreak of diarrhea were caused by highly virulent PEDV variants.

**Methods:**

A PEDV strain, YC2014, was isolated from intestinal samples of suckling piglets with acute diarrhea in 2014. The complete genomic sequence of YC2014 and the nucleotide sequence of S gene were aligned with sequences of published isolates using MEGA 5.1 software. The immune protective efficiency of YC2014 were determined by testing PEDV neutralizing antibodies in sera, the colostrum and the milk on 7th day after farrowing of the immunized sows. The diarrhea symptoms of piglets after challenge were also observed.

**Results:**

Phylogenetic analysis of the complete genomic sequence of YC2014 and the nucleotide sequence of S gene demonstrated that the YC2014 PEDV strain was clustered with the PEDV epidemic strains, with >99 % nucleotide identity to these PEDV strains. The S gene sequence of YC2014 shared only 93.9 % ~ 94.4 % identities with classical CV777, DR13 and JS2008 strains, with 15 nucleotide insertion in three sites and three nucleotide deletion in one site. The amino acid (AA) sequence of S gene of YC2014 shared only 92.8 % ~ 93.4 % identities with classical CV777, DR13 and JS2008 strains, with 5 AA insertion in two sites and 1 AA deletion in one site. In the immune protective efficiency tests, the neutralizing antibody titers in sera, the colostrum and the milk on 7th day after farrowing of the inactivated YC2014 PEDV strain immunized group were significantly higher than the inactivated CV777 immunized group and the inactivated DR13 immunized group (*P* < 0.05). The traditional inactivated PEDV vaccines made from CV777 or DR13 could not protect piglets from YC2014 challenge, while inactivated YC2014 could provide piglets with 100 % protection against YC2014 challenge.

**Conclusions:**

The results showed that, great antigenicity variation had occurred to this YC2014 PEDV strain. The YC2014 PEDV strain could provide piglets against homologous challenge. It is critical for future pathogenic and antigenic studies, as well as for the development of effective preventive and control vaccines against PEDV.

## Background

Porcine epidemic diarrhea virus (PEDV) is an enveloped, single-stranded, positive-sense RNA virus that is taxonomically classified within the family Coronaviridae, genus Alphacoronavirus. PEDV is the main causative agent of porcine epidemic diarrhea (PED), a devastating enteric disease that is characterized by watery diarrhea, vomiting, dehydration and significant mortality in piglets. Approximately 80 to 100% of PEDV-infected piglets die within 24 h of being infected with virulent PEDV strains, resulting in tremendous economic losses to the swine industry [1, 2].

Since December 2010, a large-scale outbreak of diarrhea, characterized by watery stool, dehydration, and vomiting, with 80 to 100 % morbidity and 50 to 90 % mortality in suckling piglets, has been observed in swine farms in China [3, 4]. Accumulated evidence indicates that this large-scale outbreak of diarrhea may be caused by highly virulent PEDV variants [5, 6]. In the present study, a PEDV strain, YC2014, was isolated from intestinal samples of suckling piglets with acute diarrhea in 2014, the evolutionary characteristics and the immune protective efficiency of YC2014 were also determined.

## Results

### Identification of the isolated YC2014 PEDV strain

**Fig. 1:**
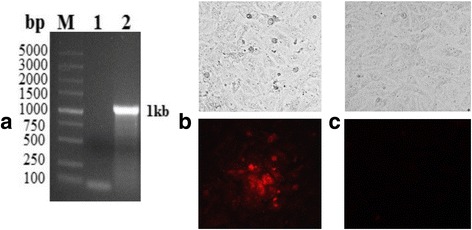
Identification of the isolated YC2014 PEDV strain. **a** RT-PCR identification of the partial gene of nucleocapsid protein. **b**, **c** Immunofluorescence assay (IFA) identification of the PEDV YC2014 strain in the cytoplasm of infected Vero cells

**Fig. 2:**
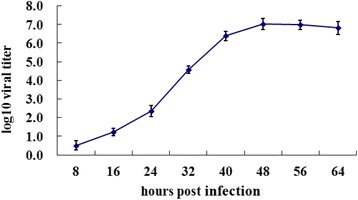
The growth kinetics study of YC2014 replicated in Vero cells

In the PEDV isolation and propagation experiment, the partial gene of nucleocapsid protein was analyzed by RT-PCR using primers N1/N2, which amplified an approximately 1 kb nucleocapsid gene fragment present in the isolated YC2014 infected Vero cells, but not in the blank control Vero cells (Fig. 1a). The isolated YC2014 PEDV strain was detected in the cytoplasm of infected Vero cells by an anti-PEDV N protein polyclonal antibody. Red fluorescence could be observed in the YC2014 strain-infected Vero cells (Fig. 1b). The isolated YC2014 PEDV strain was confirmed to be negative for other porcine enteric viruses, such as rotavirus groups A, B and C, TGEV, PRCV, calicivirus and porcine deltacoronavirus by RT-PCR. The growth kinetics study showed that YC2014 replicated rapidly and efficiently in Vero cells, reaching a maximum titer >10^7^ TCID_50_/ml by 48 hpi (Fig. 2).

### Sequence analysis of the isolated YC2014 PEDV strain

**Fig. 3:**
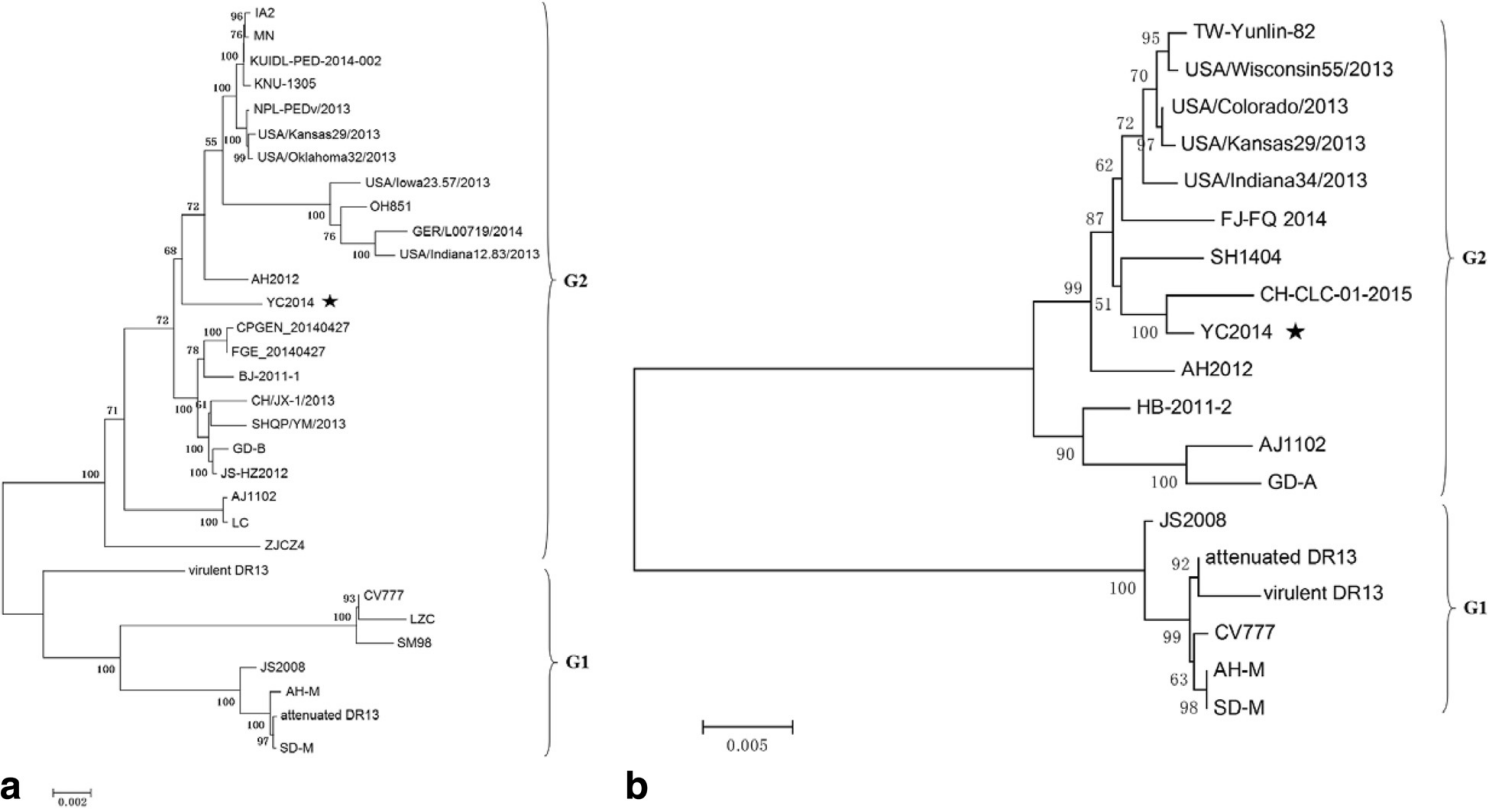
Phylogenetic analysis of the isolated YC2014 PEDV strain. **a** Phylogenetic analysis based on the complete genomic sequence of YC2014. **b** Phylogenetic analysis based on the nucleotide sequence of S gene

A total of 28,077 nucleotides were sequenced in the isolated YC2014 PEDV strain, including the polyprotein, S, ORF3, E, M, and N protein-encoding genes. The sequence of YC2014 was submitted to GenBank (accession no. KU252649). The complete genomic sequence of YC2014 and the nucleotide sequence of S gene were aligned with sequences of published isolates using MEGA 5.1. Phylogenetic analysis demonstrated that the YC2014 PEDV strain was clustered with the PEDV epidemic strains (G2 cluster, Fig. 3a and b), with >99 % nucleotide identity to these strains. The complete genome nucleotide sequence shared 96.8 % ~ 97.8 % identities with classical CV777, DR13, JS2008, AH-M and SD-M strains (G1 cluster). The S gene sequence of YC2014 shared only 93.9 % ~ 94.4 % identities with the PEDV G1 cluster, such as CV777, DR13 and JS2008 strains, with 15 nucleotide insertion in three sites (167 bp, 175 ~ 185 bp, 418 ~ 420 bp) and three nucleotide deletion in one sites (470 ~ 472 bp). The amino acid (AA) sequence of S gene of YC2014 shared only 92.8 % ~ 93.4 % identities with classical CV777, DR13 and JS2008 strains, with 5 AA insertion in two sites (AA59 ~ 62, AA140) and 1 AA deletion in one site (AA160).

### Nucleocapsid protein specific antibodies and neutralizing antibodies detection

**Fig. 4:**
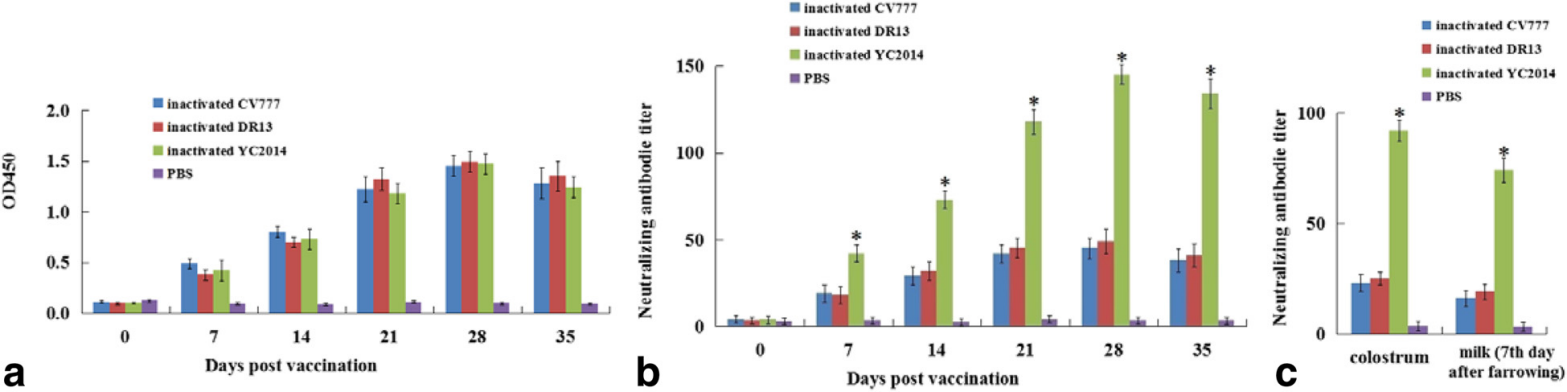
Nucleocapsid protein specific antibodies and neutralizing antibodies detection. Data were shown as mean±S.D. **a** Nucleocapsid protein specific antibodies detection in sera samples of the immunized sows. **b** Neutralizing antibodies detection in sera samples of the immunized sows. **c** Neutralizing antibodies detection in the colostrum and the milk on 7th day after farrowing. * Indicates the neutralizing antibody titer of the YC2014 PEDV strain immunized group was significantly higher than the other three groups (*P* < 0.05)

Nucleocapsid protein specific antibodies tests showed that a antibody response was detected in all the inactivated PEDV strains immunized groups 7 days after the first immunization (Fig. 4a). The antibody levels of all the inactivated PEDV strains immunized groups maintain high levels at all the testing time points, but the antibody titers of these three groups were not significantly different (*P* > 0.05). The results of PEDV neutralizing antibodies tests showed that the neutralizing antibody levels of the inactivated PEDV strains immunized groups gradually increased until 28 days after the first immunization, and still maintain high level at 7 days after farrowing. The neutralizing antibody titer in sera samples of the YC2014 PEDV strain immunized group was significantly higher than the other three groups (*P* < 0.05, Fig. 4b). The neutralizing antibody titer in the colostrum and the milk on 7th day after farrowing of the YC2014 PEDV strain immunized group was also significantly higher than the other three groups (*P* < 0.05, Fig. 4c).

### The immune protective efficiency analysis of inactivated YC2014

After YC2014 challenge, piglets in group one, group two and group four showed significant acute diarrhea. Weight gain was reduced, loss of appetite and mental uneasiness persisted, while piglets in group three, group five and group six showed no obvious diarrhea symptoms. Two days after challenge, mortality of group one, group two and group four were 100 %. No mortality or obvious clinical symptoms were observed in group three, group five and group six (Table 1).

**Table 1:** Diarrhea and mortality in piglets during virus challenge

Group	No.	Inoculation/challenge of sows	Time after challenge							
			12 h		24 h		36 h		48 h	
			Diarrhea^a^	Mortality (%)	Diarrhea	Mortality (%)	Diarrhea	Mortality (%)	Diarrhea	Mortality (%)
1	5	inactivated CV777/YC2014	+	20	++	40	++	80	++	100
2	5	inactivated DR13/YC2014	+	0	++	40	++	60	++	100
3	5	inactivated YC2014/YC2014	−	0	−	0	−	0	−	0
4	5	PBS/YC2014	+	20	++	80	++	100	++	100
5	5	inactivated CV777/CV777	−	0	−	0	−	0	−	0
6	5	inactivated DR13/DR13	−	0	−	0	−	0	−	0

^a^−, no diarrhea; +, mild diarrhea; ++, severe diarrhea

## Discussion

PED can generally be controlled using a vaccine strategy. Vaccination with killed or attenuated PEDV vaccine has been widely carried out in China and other swine raising countries, where PED usually manifests as a mild and enzootic pattern (lower mortality) some years ago. However, severe acute diarrhea outbreaks associated with high morbidity (80–100 %) and mortality (50–90 %) were observed in suckling piglets in most areas of China since December 2010, although most sow herds had previously been vaccinated with traditional inactivated PEDV vaccines based on CV777 or DR13. In April 2013, PED was diagnosed in the eastern Midwest region of the United States, subsequently, it spread rapidly to 30 neighboring states by June 2014. Accumulative evidence indicates that this large-scale outbreak of diarrhea may be caused by highly virulent PEDV variants [7–9].

The S protein makes up the large surface projections of the virion and plays a pivotal role in determining viral-cellular fusion activity and activating the immune system [10–12]. The variations in amino acid sequence likely changed the immunogenicity of the S protein and led to immunization failure of current commercial vaccines. In this study, we successfully isolated the YC2014 PEDV strain from porcine intestinal samples in dead piglets during outbreaks of acute diarrhea. The S gene nucleotides analysis of YC2014 indicated that it was clustered with the PEDV epidemic strains, with 15 nucleotide insertion in three sites and three nucleotide deletion in one site compared to classical PEDV vaccine strains CV777 and DR13. The variations of the S gene sequence and deduced amino acid sequence of the YC2014 strain compared to traditional PEDV vaccine strains may be the reason why some swine farms had well PEDV vaccine immunizations but still had sustained epidemic diarrhea which caused huge economic losses.

Vaccination is one of the most effective ways in preventing PED infection. Immunization of sows with PEDV vaccines at 20–30 days before production will provide substantial passive immunity to the newborn piglets [13]. In this study, the nucleocapsid protein specific antibody levels of three different inactivated PEDV strains immunized groups gradually increased at all the testing time points. However, the antibody titers of these three groups are not significantly different. Immunization with inactivated YC2014 could protect piglets from acute diarrhea against homologous strain challenge. Since YC2014 was clustered with the PEDV epidemic strains, with >99 % nucleotide identity to most of these epidemic strains, the inactivated YC2014 may protect other variant strains challenge in some extent. However, it needs further refine animal studies to assess this suppose. The results showed that, great antigenicity variation had occurred to this YC2014 PEDV strain under the selective pressure of vaccines. Therefore, it is important to investigate PEDV variants currently circulating in sow herds to assess their ability to allow for cross-protection against highly virulent PEDV strains and prevent PEDV epidemics.

## Conclusion

The results showed that, great antigenicity variation had occurred to this YC2014 PEDV strain. The YC2014 PEDV strain could provide piglets against homologous challenge. It is critical for future pathogenic and antigenic studies, as well as for the development of effective preventive and control vaccines against PEDV.

## Methods

### Clinical samples

In May 2014, porcine intestinal tracts, intestinal contents and fecal samples were collected from dead piglets during outbreaks of diarrhea on a breeding farm. This farm keeps more than 1800 sows and located in Yancheng city within the Jiangsu province. Clinical signs were characterized by acute vomiting, anorexia, and watery diarrhea, with high mortality in piglets less than 10 days old. The diarrhea outbreaks occurred throughout the year and re-occurred at 2–3 weeks interval.

### Virus isolation and identification

Virus isolation was performed as described previously [14]. The RNA of the isolated YC2014 PEDV strain cultures were extracted using the Viral RNA Mini kit (Geneaid Biotech, Taiwan) according to the manufacturer’s instructions. The presence of PEDV in the Vero cell culture was confirmed by reverse transcription PCR (RT-PCR) with one pairs of primers to amplify approximately 1 kb partial sequence of nucleocapsid protein, N1: GCAAACGGGTGCCATTATCTC, N2: CTAGCTCACGAACAGCCACATTAC. The samples of PEDV in the Vero cell culture were confirmed to be negative for rotavirus A, B and C, transmissible gastroenteritis virus (TGEV), porcine respiratory coronavirus (PRCV), caliciviruses and porcine deltacoronavirus via RT-PCR as previously described [15–19].

The PEDV YC2014 strain was then identified with immunofluorescence assay (IFA). Briefly, Vero cells grown on a 6-well plate were infected with the PEDV YC2014 strain. At 48 h post-infection, cells were washed twice with PBS and fixed with cold methanol for 10 min at −20 °C. Cells were then washed three times with PBST and blocked with 10 % bovine serum albumin (BSA) at 37 °C for 1 h. Preparations were incubated for 1 h at 37 °C with mouse anti-PEDV nucleocapsid protein polyclonal antibody in dilution buffer (1 % BSA in PBST), this mouse anti-PEDV nucleocapsid protein polyclonal antibody, prepared by our laboratory, was collected from serum of ICR mice immunized with purified prokaryotic expressed N protein. After three washes with PBST, cells were treated with a rhodamine-conjugated goat anti-mouse IgG (Cwbio, China) at a 1:5000 dilution with PBS for 30 min at 37 °C. After a final four washes with PBST, all wells were examined using fluorescence microscopy (Axio Observer Z1, Zeiss, Germany). After ten passages on Vero cells, the one-step growth curve of YC2014 strain in Vero cells was monitored at 8 h interval after infection.

### Sequence analysis of the PEDV YC2014 strain

Based on the sequence of the CV777 PEDV strain (GenBank: AF353511.1), seven pairs of oligonucleotide primers (Table 2) were designed to amplify the different regions of the YC2014 genome. The PCR products were cloned into the pUC19 vector using ClonExpress Entry One Step Cloning Kit (Vazyme) and sequenced by Invitrogen Biotechnology (Shanghai, China). The 5′ and 3′ ends of the genome of YC2014 were validated using the rapid amplification of cDNA ends (RACE) cDNA amplification kit (Clontech, Japan). All fragments were sequenced in both directions in triplicate. The complete genomic sequence of YC2014 and the nucleotide sequence of S gene were aligned with sequences of published isolates using MEGA 5.1 software. Phylogenetic trees were constructed using the Maximum Likelihood method and supported with a bootstrap test of 1000 replicates. Genomic sequences of the isolated YC2014 PEDV strain were submitted to GenBank under accession no. KU252649.

**Table 2:** Primers used to amplify different regions of the YC2014 genome

Primer	Sequence	Location^a^
PEDV1F	ACTTAAAAAGATTTTCTATCTACGG	1–25
PEDV1R	CGTCACTCTCGAAGAATGC	1986–2004
PEDV2F	TCTTTGAGTCTGCCTGTG	1705–1722
PEDV2R	AATTAGCATCACCATCAAATG	5008–5028
PEDV3F	CAGTTTTGCCTAATTTTGAACC	4729–4750
PEDV3R	GAGCCTACGAACTTGTCG	9011–9028
PEDV4F	CGGTGATATGTCTGTTGGC	8729–8747
PEDV4R	TAAATATCAAAATAGCGTTGCAC	14006–14028
PEDV5F	ATCATCACCAGCCCTTGTTG	13729–13748
PEDV5R	TCATTGTCAACTATAATGGCATC	20006–20028
PEDV6F	ACTTCAAGCCAGTGAATGG	19729–19747
PEDV6R	CATCTGGTAGCTGGTCGC	24332–24349
PEDV7F	GCACATTTTCTCTCTGGTAC	24042–24061
PEDV7R	GTGTATCCATATCAACACCG	28014–28033

^a^Location corresponds to position within the CV777 (AF353511.1) genome

### The immune protective efficiency analysis of inactivated YC2014

All experimental protocols were approved by the Laboratory Animal Monitoring Committee of Jiangsu province and performed accordingly. Twelve commercial high-health sows (Large White) were randomly divided into four groups (Table 3) and were housed in four separate rooms. These sows were confirmed virologically negative for PEDV, TGEV and porcine deltacoronavirus by RT-PCR and serologically negative for PEDV and TGEV antibody by indirect ELISA. On 28 and 14 days before farrowing, sows in group one were immunized intramuscularly with 2 ml of inactivated CV777 (2.0 × 10^7^ TCID_50_) emulsified in equal volume of Gel 01 ST adjuvant (Seppic, France), separately. Group two were immunized intramuscularly with 2 ml of inactivated DR13 (2.0 × 10^7^ TCID_50_) emulsified in equal volume of Gel 01 ST adjuvant. Group three were immunized intramuscularly with 2 ml of inactivated YC2014 (2.0 × 10^7^ TCID_50_) emulsified in equal volume of Gel 01 ST adjuvant. Group four were treated with 2 ml of saline emulsified in equal volume of Gel 01 ST adjuvant. After immunization, sera samples were collected from sows at 7-day intervals until 35 days after immunization (7 days after farrowing) for detection of nucleocapsid protein specific antibodies with commercial indirect ELISA kits (Biovet, Canada) and PEDV neutralizing antibodies using YC2014 as indicator virus as described previously [20]. Briefly, 50 μl of PEDV strain YC2014 (2.0 × 10^3^ TCID_50_/ml) was added to an equal volume of the sera samples and incubated for 1 h at 37 °C. The mixture was then inoculated to a 96-well plate containing confluent Vero cells. 2 h later, the culture plate was washed with D-Hank’s three times, and then added 100 μl of DMEM containing 2 % fetal bovine serum (FBS). 48 h later, the culture plate was fixed with cold methanol for 10 min at −20 °C, and incubated with mouse anti-PEDV nucleocapsid protein polyclonal antibody for 1 h at 37 °C, and then stained with FITC-labeled rabbit anti-mouse IgG (Santa Cruz Biotechnology). The serum titers were determined as the reciprocal of the last serum dilution at 70 % or greater fluorescent focus reduction in the infected cell cultures under a fluorescent microscope. The colostrum and the milk on 7th day after farrowing of each sow were also collected and used for the detection of PEDV neutralizing antibodies.

**Table 3:** Inoculation of the sows

Group	No.	Immunogen	Dose
1	3	inactivated CV777	2.0 × 10^7^ TCID_50_
2	3	inactivated DR13	2.0 × 10^7^ TCID_50_
3	3	inactivated YC2014	2.0 × 10^7^ TCID_50_
4	3	PBS	----

The average number of sows farrowing was 13, piglets in each group was coded and chosen randomly, respectively. On 7th day after farrowing, thirty piglets were chosen (Table 4), ten from group one (inactivated CV777 immunized group), ten from group two (inactivated DR13 immunized group), five from group three (inactivated YC2014 immunized group), and five from group four (saline treated group). These piglets were divided into six groups (Table 4), each group were housed in a separate room and were artificial feeding with milk. Piglets in group one to group four were challenged orally with 1.0 × 10^4^ TCID_50_ of YC2014. Group five were challenged orally with 1.0 × 10^4^ TCID_50_ of CV777. Group six were challenged orally with 1.0 × 10^4^ TCID_50_ of DR13. Piglets were observed daily after challenge about the diarrhea symptoms.

**Table 4:** Challenge of the piglets

Group	No.	Inoculation of the sows	Challenge
1	5	inactivated CV777	YC2014, 1.0 × 10^4^ TCID_50_
2	5	inactivated DR13	
3	5	inactivated YC2014	
4	5	PBS	
5	5	inactivated CV777	CV777, 1.0 × 10^4^ TCID_50_
6	5	inactivated DR13	DR13, 1.0 × 10^4^ TCID_50_

### Statistical analysis

All data were analyzed using one-way ANOVA and values of *P* < 0.05 were considered statistically significant.
